# Alert-associated cardiovascular events in Gulf civilian populations: emerging risks and surveillance opportunities

**DOI:** 10.3389/fpubh.2026.1843211

**Published:** 2026-06-18

**Authors:** Hassa Iftikhar

**Affiliations:** Department of Cardiology, Tongji Medical College, Huazhong University of Science and Technology, Hubei, Wuhan, China

**Keywords:** acute coronary syndromes, cardiovascular disease, digital health surveillance, migrant health, public health preparedness

## Abstract

Conflict and disaster settings are increasingly recognized as triggers for acute cardiovascular events; however, little attention has been given to civilian populations exposed to intermittent conflict alerts in non-war environments. This Perspective highlights the potential for alert-associated cardiovascular events, including overt myocardial infarction and subclinical myocardial injury, in Gulf host populations with high baseline cardiometabolic risk. Drawing on evidence from conflict and disaster literature, we propose that acute stress responses triggered by missile alerts or security warnings may precipitate arrhythmias and acute coronary syndromes within short temporal windows. We further argue that current surveillance systems are not designed to detect such transient events due to the lack of integration between alert timelines and cardiovascular data. The Gulf’s advanced digital health infrastructure presents a unique opportunity to develop time-linked cardiovascular monitoring through the integration of civil defence alerts, hospital registries, and wearable technologies. Addressing this gap could transform an under-recognized cardiovascular risk into a measurable and preventable component of public health preparedness. Future research should focus on defining diagnostic criteria, improving data integration, and ensuring equitable inclusion of migrant populations.

## Introduction

In early 2024, escalating Iran-US tensions and missile threats across the Gulf illustrated how “near-war” events can expose civilian populations to fear and disruption without escalating into large-scale war conditions. The Eastern Mediterranean region has among the highest cardiovascular mortality rates globally, particularly driven by cardiometabolic risk in populations under the age of 60 (1). Evidence from conflict and disaster settings demonstrates an increased incidence of acute coronary syndromes, arrhythmias, and stroke in affected civilian populations, as shown in systematic analyses of conflict-affected populations (2). However, few studies have examined Gulf host states such as the UAE, and almost none investigated short-term cardiovascular responses to conflict alerts or missile warnings outside battlefields.

This persective argues that conflict alerts may precipitate both overt and subclinical cardiac events in high-risk Gulf civilians; however, existing surveillance systems and research frameworks are poorly equipped to detect these events. We define alert-associated myocardial injury as overt myocardial infarction or subclinical injury, such as elevated cardiac troponin levels without classical symptoms which may remain unrecognized in routine surveillance (3). However, standardized diagnostic thresholds and temporal windows for identifying subclinical myocardial injury following acute conflict alerts have not yet been established, representing a key limitation for future research. Systematic reviews highlighted the biological plausibility of stress-related surges in blood pressure, heart rate, and psychological distress (1). Regional cardiovascular research priorities still focus mainly on prevention strategies, service delivery, and risk prediction tools rather than conflict-related- triggers or civil-defence scenarios (2).

Framing these risks as physiological curiosities overlooks their implications for public health. Conflict alerts intersect with the already high burden of cardiovascular disease in the region, creating a significant health blind spot. High-risk-subgroups, including migrant workers, low-income- residents, and younger adults with cardiometabolic risk, may experience acute physiological stress during alerts, potentially triggering acute cardiovascular stress responses (1, 2). However, fragmented health surveillance, access barriers, and the absence of conflict-sensitive cardiovascular policies limit the recognition of these events in the literature. Integrating conflict alerts into cardiovascular preparedness is essential for non-communicable disease control and civilian protection in the Gulf. This Perspective highlight current evidence, identifies key gaps, and proposes a framework for integrating conflict alert timelines into cardiovascular surveillance systems.

### Host populations and migrants at the edge of the data

Most existing war-cardiology evidence is drawn from traditional, high-intensity- conflict zones and displaced populations, such as Bosnia, Croatia, Colombia, Syria, and Ukraine. Jawad and colleagues’ systematic review of armed conflict and cardiovascular risk identified 65 studies across 23 conflicts and noted that the literature is heavily concentrated in a small number of settings outside the Gulf while conflicts within the Middle East remain strikingly under-represented (1, 3, 4). Supplementary Table S1 summarizes the key study domains from the literature and highlights the remaining evidence gaps relevant to Gulf host and migrant civilian populations.

In contrast, civilians in conflict-exposed- states, such as the UAE and other GCC countries, remain largely absent from the evidence base. Simultaneously, large migrant worker communities in the manual labor and service sectors face hazardous working conditions, limited preventive care, and significant occupational stress. Migrant workers in GCC countries experience physical and psychological morbidity with constrained healthcare access, making them susceptible to stress-related cardiac events that may never receive clinical attention (5). These structural vulnerabilities increase the likelihood that subclinical or unrecognized episodes remain undetected during routine hospital surveillance.

Regional analyses and UAE-specific- studies have shown substantial cardiometabolic risk among relatively young adults, with prevalence varying by sex, nationality, education, and behavioral factors. Premature myocardial infarction is common in the Middle East and North Africa, often affecting younger men who smoke heavily. However, the interaction between this high baseline cardiovascular risk and conflict alert- stressors, including sudden fear of attack, disruption of work and housing, and separation from family, remains poorly understood. No studies have examined whether repeated alerts differentially impact cardiovascular events in women versus men, Gulf nationals versus migrants, or across ethnic groups, despite calls for sex-stratified- conflict cardiovascular analyses (1, 5). Conducting such stratified analyses in Gulf settings may also face practical challenges, including the limited availability of granular demographic variables in existing registries, privacy considerations surrounding migrant populations, and linguistic or cultural barriers to community-based data collection. However, Gulf host populations, particularly migrant workers, remain largely absent from the conflict-cardiovascular literature. The evidence linking conflict alerts to myocardial infarction or arrhythmias in Gulf remains limited.

### Myocardial injury and arrhythmias in the ‘alert window’

Evidence from disasters and conflict events shows short-term- spikes in ST-elevation- myocardial infarction (STEMI), acute coronary syndromes (ACS), and arrhythmias immediately after intense stress events. Most analyses aggregate outcomes over days or weeks rather than aligning events with precise alert windows, such as sirens or emergency notifications (2). Table 1 summarizes the current evidence from non-Gulf settings, key knowledge gaps in Gulf host populations, and implications for cardiovascular disease detection and equity. This leaves two critical research questions for civilian populations in the Gulf:

1. Are the incidence rates of arrhythmias, myocardial infarction, or acute coronary syndromes increased within hours of conflict alerts or explosions?
2. What proportion represents subclinical myocardial injury or unrecognized myocardial infarction that never reaches the hospital?

**Table 1:** Current evidence and knowledge gaps on conflict-alert-associated cardiovascular events in Gulf civilian populations.

Domain	What is known (primarily from non-Gulf settings)	What is unknown in Gulf host populations	Implications for equity and detection
Sleep disruption and nocturnal alerts	Sleep disturbance and circadian disruption are associated with increased cardiovascular risk, including blood pressure variability, autonomic imbalance, and higher risk of nocturnal cardiac events	The cardiovascular impact of repeated nocturnal conflict alerts or sirens has not been examined in Gulf civilian populations	Night-time alert systems may unintentionally create high-risk cardiovascular windows, particularly for individuals with existing cardiovascular disease
Acute myocardial infarction/acute coronary syndromes	Previous studies from conflict regions and disaster settings have reported increases in acute coronary syndromes following missile attacks, terror events, and major disasters, likely triggered by acute psychological stress and sympathetic activation	There are no high-resolution temporal studies examining whether myocardial infarction incidence increases within hours of missile alerts or explosions in Gulf civilian populations	Potential short-term spikes in acute coronary events may remain unrecognized without time-linked surveillance, leading to missed opportunities for early detection and emergency preparedness
Arrhythmias and sudden cardiac events	Acute emotional stress and environmental shocks have been associated with increased arrhythmogenic risk, including atrial fibrillation, ventricular arrhythmias, and sudden cardiac death in disaster contexts	The relationship between conflict alerts and arrhythmia incidence has not been systematically evaluated in Gulf civilian populations, particularly using wearable or digital monitoring tools	Lack of real-time monitoring may obscure transient arrhythmic events triggered by conflict-related stress, among individuals with pre-existing cardiovascular disease
Takotsubo cardiomyopathy	Stress-induced cardiomyopathy (Takotsubo syndrome) has been reported following intense emotional stress during disasters and conflict events	Conflict-alert-associated Takotsubo events have not been evaluated in Gulf populations	Stress-induced cardiac injury related to acute emotional stress may remain under-recognized in emergency settings
Silent myocardial injury/subclinical ischemia	Silent myocardial infarction and subclinical myocardial injury are increasingly recognized through biomarker studies and imaging in high-risk populations	The potential for conflict alerts to precipitate silent myocardial injury that does not reach hospital care has not been studied in Gulf civilian or migrant populations	Subclinical cardiac events may remain undetected, especially among populations with limited healthcare access or barriers to seeking medical care
Migrant and under-documented populations	Migrant populations globally often experience higher cardiovascular risk due to occupational stress, limited healthcare access, and socioeconomic barriers	The burden of acute or silent cardiovascular events during conflict alerts among migrant workers in Gulf countries remains largely undocumented	Under-representation of migrant populations in surveillance systems may exacerbate cardio-equity gaps and obscure the true population burden
Digital cardiovascular surveillance	Wearable devices and digital health tools can detect arrhythmias, heart rate variability changes, and early signs of cardiovascular stress in real time	No integrated systems currently link conflict alert timing with digital cardiovascular monitoring or hospital surveillance in Gulf countries	Failure to leverage digital infrastructure may result in missed opportunities for early detection of conflict-related cardiovascular risk in highly connected populations

The biological plausibility is strong: acute catecholamine surges, blood pressure spikes, endothelial dysfunction, and myocardial ischemia are established pathways for coronary events and arrhythmias; however, none have been examined in relation to conflict alerts in Gulf populations. Another major gap is the absence of high-resolution temporal analyses linking the exact timing of alerts with cardiovascular outcomes, leaving the short “alert window” (minutes to hours following alerts) for cardiac risk largely unmapped (4).

Nocturnal alerts represent a particularly underexplored high-risk window. Nighttime sirens, sudden missile-related- notifications, or explosions that fragment sleep may interact with circadian peaks in sympathetic activity, blood pressure variability, and prothrombotic tendencies, amplifying the risk of ACS and malignant arrhythmias (2, 6). However, no study has systematically tracked nocturnal cardiovascular events, troponin rises, or arrhythmia episodes triggered by missile alerts, sirens, or security warnings. The Gulf’s advanced digital infrastructure presents a unique opportunity to achieve this precision through integrated surveillance approaches that have not yet been implemented worldwide.

### Digital Gulf, analogue surveillance

**Figure 1:**
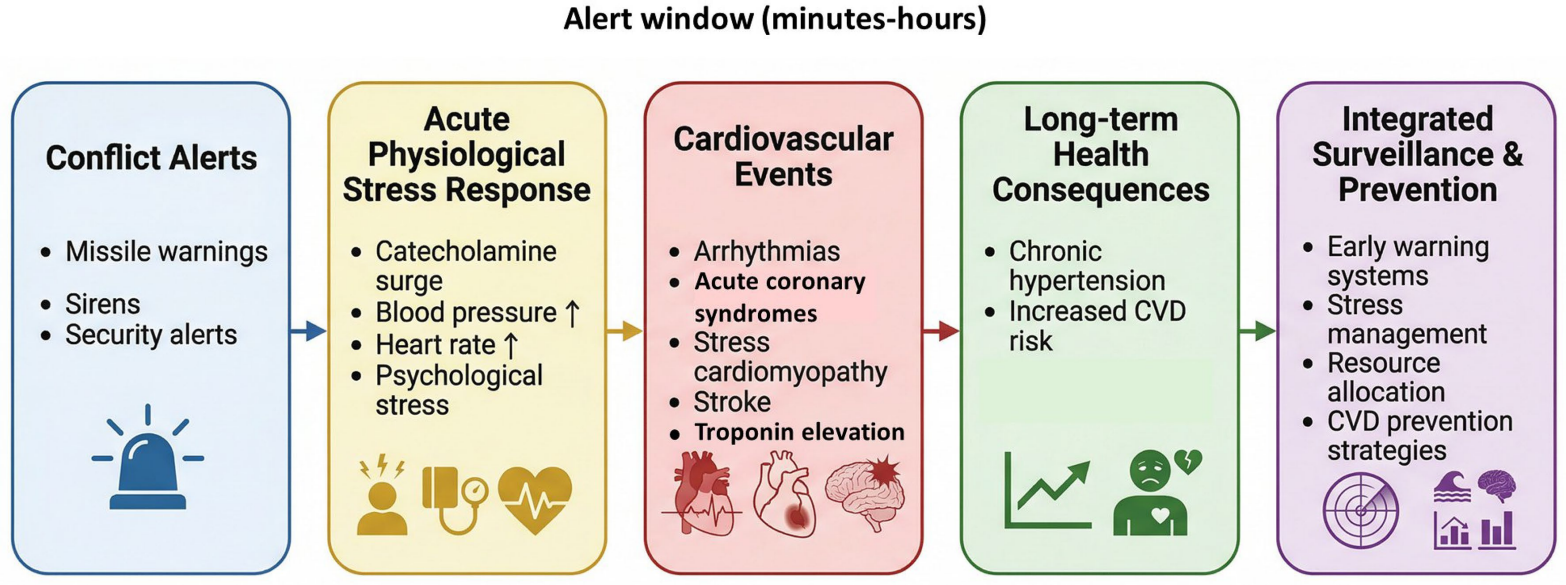
Conceptual framework linking conflict alerts to acute cardiovascular events and integrated surveillance strategies in Gulf populations. Conflict alerts such as missile warnings and sirens may trigger acute physiological stress responses, including catecholamine surges and increases in blood pressure and heart rate. These responses may precipitate acute cardiovascular events, including arrhythmias, acute coronary syndromes, and subclinical myocardial injury. Current surveillance systems lack temporal integration with alert timelines, limiting detection of transient events occurring within short “alert windows.” Integration of civil defence alert systems with hospital registries and wearable health technologies could enable real-time detection, risk stratification, and improved cardiovascular preparedness in Gulf populations.

The Gulf is highly digitally connected, with widespread use of smartphones and wearable devices capable of photoplethysmography (PPG), heart rate monitoring, and basic ECG recording. Despite this digital capacity, surveillance systems rarely link conflict alert timelines to cardiovascular outcomes, no integrated platforms currently link alert timing with cardiovascular outcomes (7). Consequently, transient cardiac events occurring during or shortly after alerts may remain undetected in routine hospital reporting systems. The Gulf’s advanced digital health infrastructure presents a significant opportunity to address this gap. Emerging digital health infrastructure could enable time-linked monitoring of cardiovascular responses to alerts by integrating civil defence alerts, hospital registries, and wearable data. Such approaches can detect clusters of myocardial injury or arrhythmias following acute stress events (2). A conceptual framework illustrating the pathway from conflict alerts to cardiovascular events, existing surveillance gaps, and proposed integrated monitoring strategies is presented in Figure 1. A key priority is integrating timelines of recent alerts and explosions with cardiovascular admissions and deaths in UAE and regional hospitals, stratified by nationality, sex, age, and occupation. Second, no protocols exist for using wearable ECG/PPG, heart rate- variability, or home blood pressure monitoring around alert periods in high-risk individuals or for remote troponin testing, despite rapid advances in digital cardiology (8). Third, community-level post-alert cardiovascular screening is rarely implemented particularly among populations facing barriers to healthcare access including migrant workers (1, 6). However, privacy, feasibility, and acceptability issues must be addressed. Future digital cardiovascular monitoring initiatives will require robust ethical governance frameworks, including data anonymization, secure data storage, and independent oversight mechanisms. Surveillance fears, potential data misuse, and employment consequences may discourage migrant workers from participating in studies. Transparent consent processes and collaboration with worker communities will be essential to ensure participation and prevent misuse of health data (1, 5, 6).

## Discussion

Addressing these gaps requires coordinated research and policy initiatives within the Gulf health system. However, implementation may face practical barriers, including coordination between civil defence and health authorities, resource allocation for new surveillance infrastructure, and sustained institutional support. First, the timeline of recent alerts and explosions should be associated with cardiovascular admissions and deaths in the UAE and regional hospitals, stratified by nationality, sex, age, and occupation (2, 6). Second, prospective digitally enabled cohorts of high-risk patients such as coronary artery disease or diabetes could share wearable data and symptom alerts, with targeted post-alert ECG and troponin measurements to detect subclinical myocardial injury (1, 2, 9). Third, migrant-focused- outreach and screening programs, including mobile clinics or pop-up- services within 24–72 h after alerts, can identify community-level events (5). Furthermore, integrating cardiovascular prompts into national alert systems, such as symptom checklists, medication guidance, and remote triage, could strengthen the emergency cardiac response (2). These actions align with the efforts to strengthen health systems and promote equity in populations facing chronic non-communicable disease burdens (1, 2, 9).

## Limitation

This perspective has several limitations. It is conceptual and policy-oriented and does not include primary data collection or statistical analysis of the cardiovascular events. The discussion focuses primarily on Gulf host states and does not provide a comprehensive global comparison of surveillance approaches in other conflict-exposed or disaster-prone regions. Methodological challenges remain for future research, including defining diagnostic thresholds for subclinical myocardial injury following alerts and ensuring equitable data collection among migrant populations. These limitations highlight the need for prospective studies and pilot surveillance programs to validate the hypotheses proposed in this study. The main aim is to highlight emerging research gaps rather than provide a comprehensive methodological framework.

## Conclusion

Existing conflict-CVD evidence, emergency care- gaps, and regional NCD burdens suggest that alert-linked- myocardial injury and arrhythmias are plausible but poorly detected within current surveillance systems. Harnessing the Gulf’s advanced digital infrastructure through time-locked- cardiovascular monitoring and targeted outreach offers a realistic pathway for identifying these events in the future. Such approaches could transform an unrecognized cardiovascular risks into measurable components of civilian conflict preparedness while strengthening health equity.

## Data Availability

The original contributions presented in the study are included in the article/supplementary material, further inquiries can be directed to the corresponding author.
