# Supplementary material for: Alert-associated cardiovascular events in Gulf civilian populations: emerging risks and surveillance opportunities
Source: Front Public Health. 2026 Jun 18;14:1843211. doi: 10.3389/fpubh.2026.1843211 (PMC13323006; doi:10.3389/fpubh.2026.1843211)
Supplement: Supplementary file 1 [file Supplementary_file_1.pptx]

## Slide 1
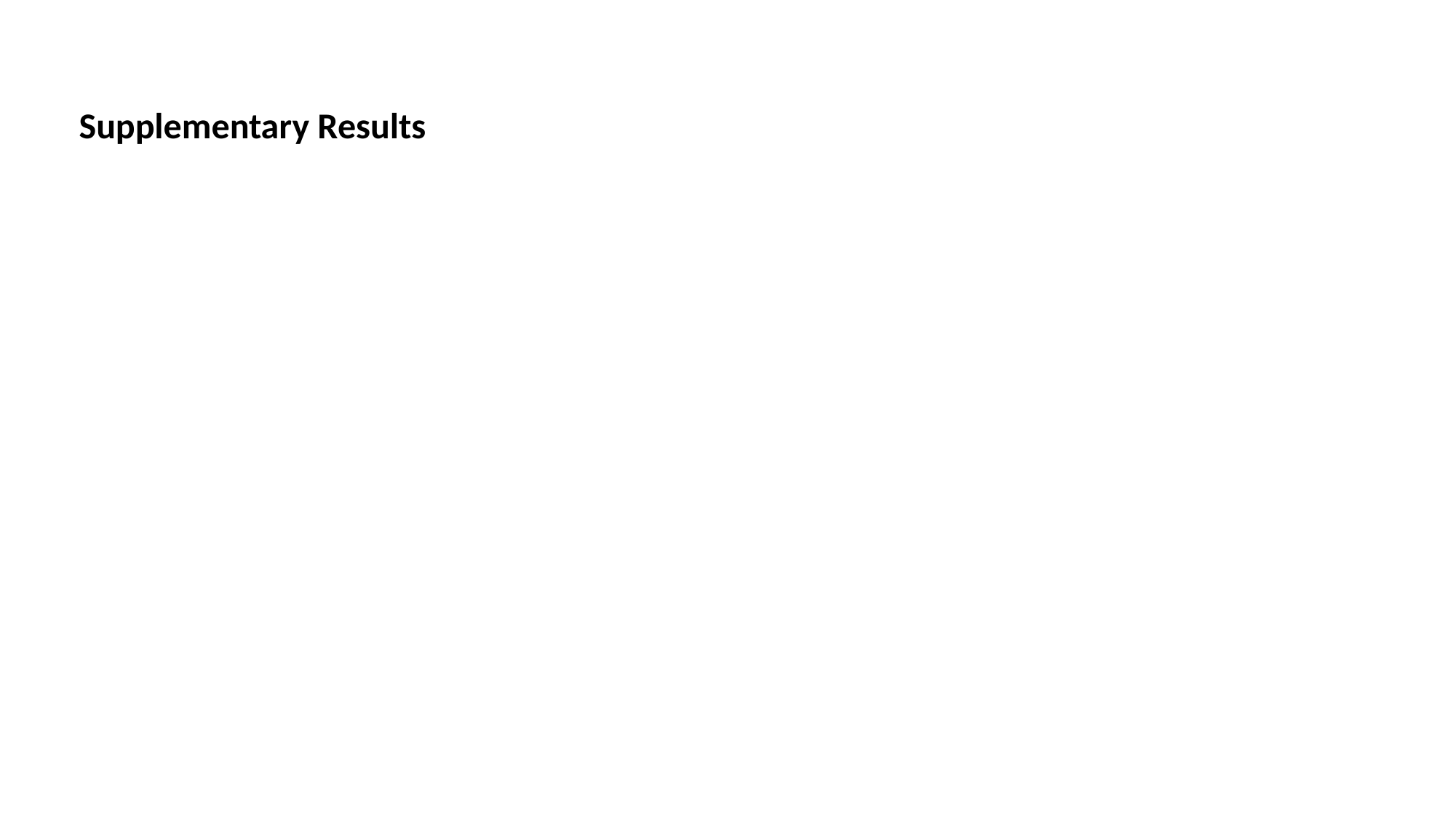

Supplementary Results

## Slide 2
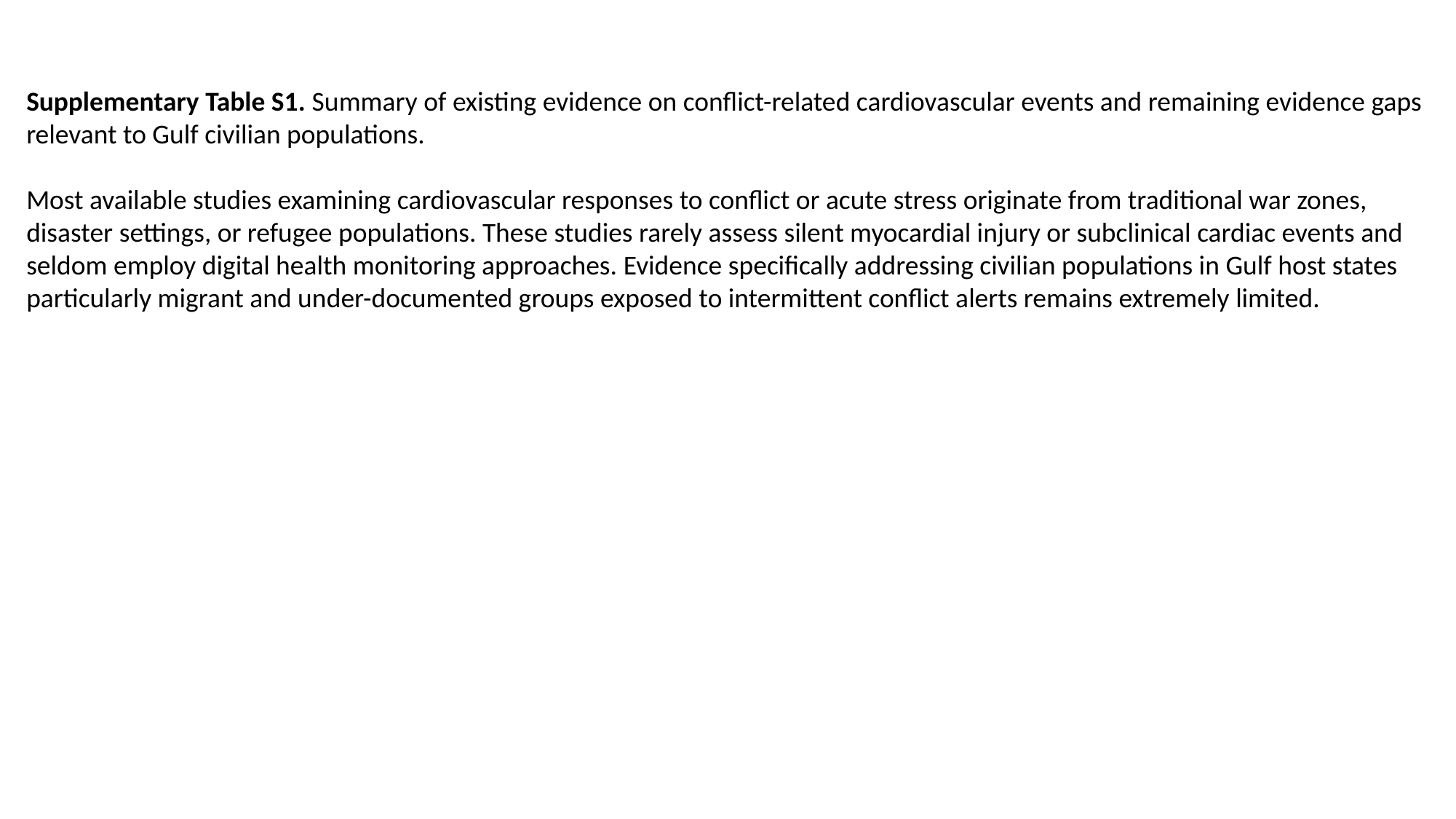

Supplementary Table S1. Summary of existing evidence on conflict-related cardiovascular events and remaining evidence gaps relevant to Gulf civilian populations.
Most available studies examining cardiovascular responses to conflict or acute stress originate from traditional war zones, disaster settings, or refugee populations. These studies rarely assess silent myocardial injury or subclinical cardiac events and seldom employ digital health monitoring approaches. Evidence specifically addressing civilian populations in Gulf host states particularly migrant and under-documented groups exposed to intermittent conflict alerts remains extremely limited.

## Slide 3
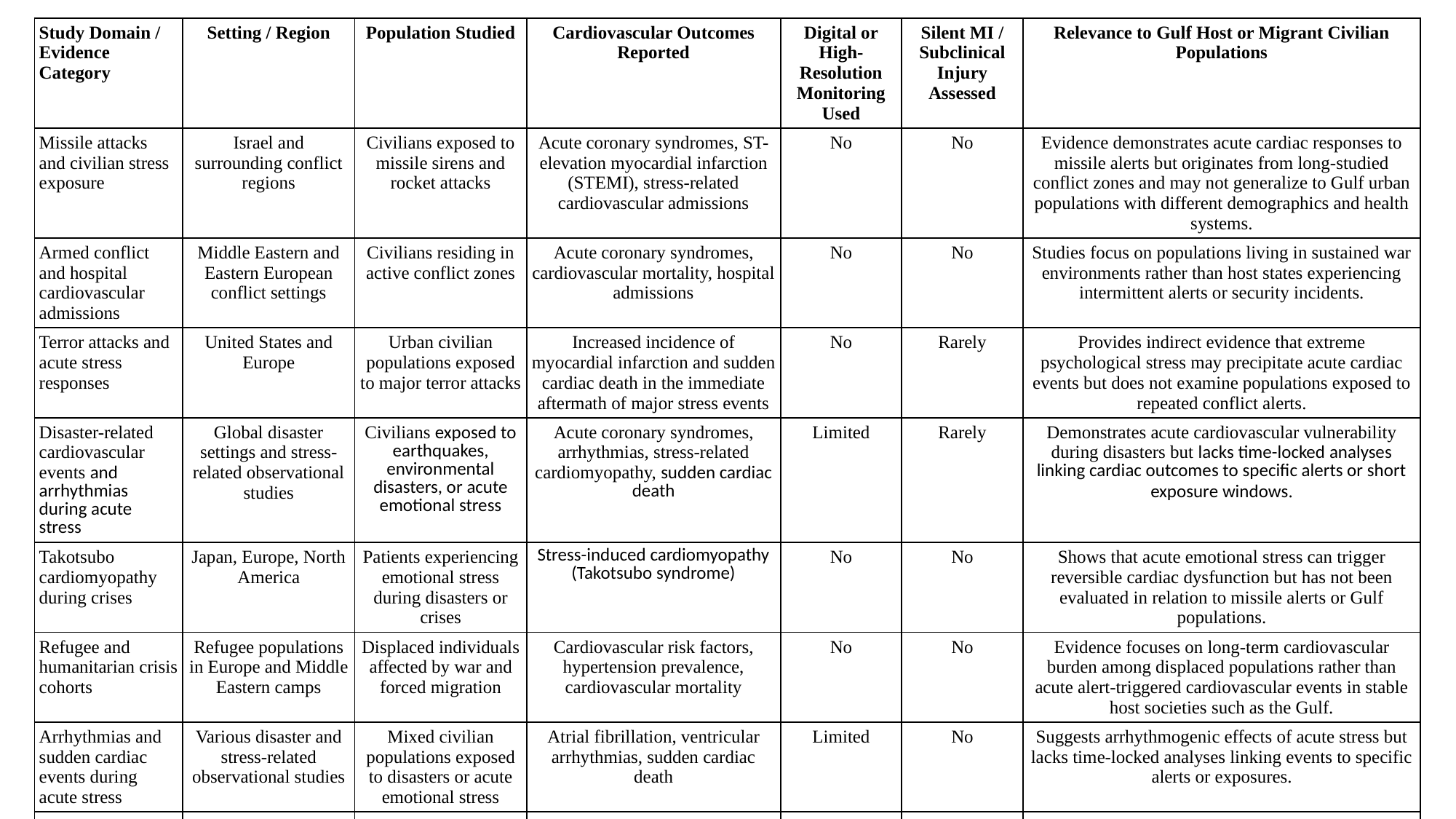

| Study Domain / Evidence Category | Setting / Region | Population Studied | Cardiovascular Outcomes Reported | Digital or High-Resolution Monitoring Used | Silent MI / Subclinical Injury Assessed | Relevance to Gulf Host or Migrant Civilian Populations |
| --- | --- | --- | --- | --- | --- | --- |
| Missile attacks and civilian stress exposure | Israel and surrounding conflict regions | Civilians exposed to missile sirens and rocket attacks | Acute coronary syndromes, ST-elevation myocardial infarction (STEMI), stress-related cardiovascular admissions | No | No | Evidence demonstrates acute cardiac responses to missile alerts but originates from long-studied conflict zones and may not generalize to Gulf urban populations with different demographics and health systems. |
| Armed conflict and hospital cardiovascular admissions | Middle Eastern and Eastern European conflict settings | Civilians residing in active conflict zones | Acute coronary syndromes, cardiovascular mortality, hospital admissions | No | No | Studies focus on populations living in sustained war environments rather than host states experiencing intermittent alerts or security incidents. |
| Terror attacks and acute stress responses | United States and Europe | Urban civilian populations exposed to major terror attacks | Increased incidence of myocardial infarction and sudden cardiac death in the immediate aftermath of major stress events | No | Rarely | Provides indirect evidence that extreme psychological stress may precipitate acute cardiac events but does not examine populations exposed to repeated conflict alerts. |
| Disaster-related cardiovascular events and arrhythmias during acute stress | Global disaster settings and stress-related observational studies | Civilians exposed to earthquakes, environmental disasters, or acute emotional stress | Acute coronary syndromes, arrhythmias, stress-related cardiomyopathy, sudden cardiac death | Limited | Rarely | Demonstrates acute cardiovascular vulnerability during disasters but lacks time-locked analyses linking cardiac outcomes to specific alerts or short exposure windows. |
| Takotsubo cardiomyopathy during crises | Japan, Europe, North America | Patients experiencing emotional stress during disasters or crises | Stress-induced cardiomyopathy (Takotsubo syndrome) | No | No | Shows that acute emotional stress can trigger reversible cardiac dysfunction but has not been evaluated in relation to missile alerts or Gulf populations. |
| Refugee and humanitarian crisis cohorts | Refugee populations in Europe and Middle Eastern camps | Displaced individuals affected by war and forced migration | Cardiovascular risk factors, hypertension prevalence, cardiovascular mortality | No | No | Evidence focuses on long-term cardiovascular burden among displaced populations rather than acute alert-triggered cardiovascular events in stable host societies such as the Gulf. |
| Arrhythmias and sudden cardiac events during acute stress | Various disaster and stress-related observational studies | Mixed civilian populations exposed to disasters or acute emotional stress | Atrial fibrillation, ventricular arrhythmias, sudden cardiac death | Limited | No | Suggests arrhythmogenic effects of acute stress but lacks time-locked analyses linking events to specific alerts or exposures. |
| Digital cardiovascular monitoring in population health | High-income countries using wearable devices and remote monitoring | Community cohorts using wearable or smartphone health devices | Detection of arrhythmias, heart-rate variability changes, and early cardiovascular abnormalities | Yes | Occasionally | Demonstrates the potential for real-time cardiovascular monitoring but has not been applied to conflict-alert settings or integrated with emergency alert systems. |
